# Desiccation Tolerance as the Basis of Long-Term Seed Viability

**DOI:** 10.3390/ijms22010101

**Published:** 2020-12-24

**Authors:** Galina Smolikova, Tatiana Leonova, Natalia Vashurina, Andrej Frolov, Sergei Medvedev

**Affiliations:** 1 Department of Plant Physiology and Biochemistry, St. Petersburg State University, 199034 St. Petersburg, Russia; s.medvedev@spbu.ru; 2 Department of Biochemistry, St. Petersburg State University, 199004 St. Petersburg, Russia; tanyaleonova2710@gmail.com (T.L.); vashurina.nat@yandex.ru (N.V.); afrolov@ipb-halle.de (A.F.); 3 Department of Bioorganic Chemistry, Leibniz Institute of Plant Biochemistry, 06120 Halle (Saale), Germany

**Keywords:** abscisic acid, after-ripening, desiccation tolerance, dormancy, germination, gibberellins, LAFL, seeds, viability

## Abstract

Desiccation tolerance appeared as the key adaptation feature of photoautotrophic organisms for survival in terrestrial habitats. During the further evolution, vascular plants developed complex anatomy structures and molecular mechanisms to maintain the hydrated state of cell environment and sustain dehydration. However, the role of the genes encoding the mechanisms behind this adaptive feature of terrestrial plants changed with their evolution. Thus, in higher vascular plants it is restricted to protection of spores, seeds and pollen from dehydration, whereas the mature vegetative stages became sensitive to desiccation. During maturation, orthodox seeds lose up to 95% of water and successfully enter dormancy. This feature allows seeds maintaining their viability even under strongly fluctuating environmental conditions. The mechanisms behind the desiccation tolerance are activated at the late seed maturation stage and are associated with the accumulation of late embryogenesis abundant (LEA) proteins, small heat shock proteins (sHSP), non-reducing oligosaccharides, and antioxidants of different chemical nature. The main regulators of maturation and desiccation tolerance are abscisic acid and protein DOG1, which control the network of transcription factors, represented by LEC1, LEC2, FUS3, ABI3, ABI5, AGL67, PLATZ1, PLATZ2. This network is complemented by epigenetic regulation of gene expression via methylation of DNA, post-translational modifications of histones and chromatin remodeling. These fine regulatory mechanisms allow orthodox seeds maintaining desiccation tolerance during the whole period of germination up to the stage of radicle protrusion. This time point, in which seeds lose desiccation tolerance, is critical for the whole process of seed development.

## 1. Introduction 

The ability to sustain dehydration and survive during desiccation appeared as a crucial evolutionary step, which allowed the first plants colonizing the terrestrial habitat [1]. Rapid mobilization of tolerance mechanisms during desiccation and subsequent re-hydration is common for so-called poikilohydric plants—bryophytes, some algae and ferns, as well as a small group of angiosperm plants [2]. Although this feature was mostly lost during the further evolution of terrestrial flora, more than 60 species of spore vascular plants and about 140 species of angiosperms are known to be tolerant to desiccation [3]. However, due to their slow growth, such plants have limited geographic distribution. Phylogenesis of seed plants is characterized by continuously increasing complexity of anatomy structures, which, on one hand, essentially restrict water loss from the plant surface (cuticle, endoderm, stomata), on the other—makes its transport within the plant more efficient (tracheary elements) [1,2]. Unfortunately, the restriction in water penetration across the plant surface barrier structures resulted in a limitation of its transport in both directions, i.e., the enhanced water retaining capability turned to be accompanied with compromised viability under severe dehydration conditions. Thus, the role of the genes, which encoded adaptation of the whole organism to water loss at earlier steps of plant evolution, in higher vascular plants is restricted to the protection of spores, seeds and pollen from dehydration. This fact represents a principal ecological paradox: the mature plants, which are sensitive to even minimal dehydration and dramatically reduce their productivity under drought conditions, are able to tolerate almost complete loss of water without noticeable damage at the stage of seed.

In respect to desiccation tolerance (DT), the seeds of vascular plants can be classified into orthodox and recalcitrant types [4,5,6]. The maturation of the orthodox seeds is accompanied with a water loss up to 5–10% *w/w*, which allows them sustaining unfavorable environmental conditions, such as extremely high and low temperatures and drought. In contrast, water loss during maturation is not characteristic for recalcitrant seeds. These seeds are sensitive to dehydration and, similarly to the tissues of mature vascular plants, are damaged during strong desiccation. Thus, the viability of dormant recalcitrant seeds is highly dependent on environmental conditions. Due to this fact, their distribution is restricted to tropical forests. Some authors believe that the plant species with recalcitrant seeds evolved from ancestors with orthodox seeds as a result of adaptation to wet climate with constant rains, where immediate germination upon separation from the parental plant would be evolutionary advantageous [1]. Besides these two contrasting seed development strategies, so-called “intermediate” plant species, the mature seeds of which are able to sustain slight dehydration, are also known [1,5,6]. The differences in dehydration tolerance of orthodox and recalcitrant seeds are important for the long-term storage in gene banks [7].

In orthodox seeds, the mechanisms behind the onset of desiccation tolerance are activated at the final stages of maturation [8]. Later on, desiccation tolerance is lost during germination, at the moment of radicle protrusion [4,9]. Although the phenomenon of drought tolerance is being intensively studied in plants, the molecular mechanisms behind the onset and loss of desiccation tolerance during seed maturation and germination, respectively, remain mostly unknown. To some extent, these mechanisms can be approached by deeper evaluation of the molecular events behind the physiological and biochemical adaptation in so-called resurrection plants, which acquired desiccation tolerance as a secondary adaptation during the evolution, and, therefore, do not lose the viability of vegetative organs after drying/rehydration cycles [3,10]. Resurrection plants are generally small and can grow on soils with a thin fertile layer (e.g., on rock outcrops). It is assumed that the molecular mechanisms underlying desiccation tolerance of resurrection plants and mature orthodox seeds are essentially similar [4].

## 2. Mechanisms behind the Onset of Desiccation Tolerance during Seed Maturation

**Figure 1:**
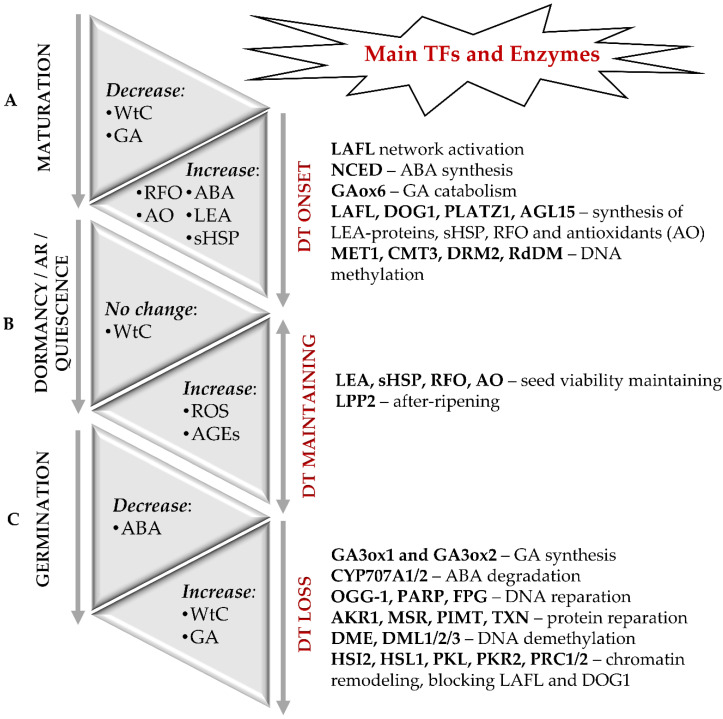
Molecular events accompanying the onset and loss of desiccation tolerance (DT) in orthodox seeds. (**A**) The onset of DT and seed dormancy is defined during the late maturation stage and associated with a sharp decrease in water content (WtC). The molecular basis of DT is the accumulation of LEA proteins, sHSP, RFO, and antioxidants. Regulation of seed maturation relies on the balance of ABA and GAs along with LAFL—the network of the transcription factors LEC1, ABI3, FUS3 and LEC2, as well as by DOG1, DOG4 and DOGL4 proteins. In addition to the transcription factors of LAFL network as major regulators of desiccation tolerance, PLATZ 1, PLATZ2, and AGL67 can be also involved. Epigenetic modifications, such as changes in the patterns of DNA methylation and histone post-translational modifications are also crucial for the onset of DT. (**B**) Long-term storage can result in enhanced damage of dormant seeds due to non-enzymatic modifications of proteins with ROS and glycation agents. After-ripening (AR) results in termination of dormancy and switch of seeds to a quiescent state, in which they can germinate when favorable environmental conditions occur. (**C**) The transition from the quiescent state to germination is associated with rapid water uptake, hydration of macromolecules, repair of membranes, proteins, and nuclear acids. It is accompanied with enhanced generation of H_2_O_2_ as a secondary messenger. Activity of the catabolic enzymes involved in the oxidative degradation of ABA and the level of major active GA increase. The LAFL-controlled program gets blocked at the moment of radicle protrusion, and seeds lose desiccation tolerance. Modifications of chromatin, which involve PRC1 and PRC2, as well as PKL and PKR2 proteins, represent the key mechanism behind the LAFL suppression.

In general, seed formation includes two principal sequential steps—development of embryo and seed maturation. Thereby, seed maturation can be sub-divided in early, middle and late maturation stages. During the embryo development, its axial structures are formed, tissue-specific cell differentiation starts, and the main plant organs are defined [11,12]. During the early seed maturation stage, storage compounds (proteins, fats, and carbohydrates) are intensively accumulated [13]. At the late seed maturation stage, transition to dormancy and onset of desiccation tolerance occur [8]. The molecular basis of the desiccation tolerance of orthodox seeds is accumulation of protectors—late embryogenesis abundant (LEA) proteins, small heat shock proteins (sHSP), non-reducing oligosaccharides of the raffinose group (RFO) [1,3,8,14] and low molecular weight antioxidants—glutathione [15], tocopherols [16] and carotenoids [17] (Figure 1A).

### 2.1. Late Embryogenesis Abundant (LEA) Proteins

LEA proteins play the key role in sustaining of seed viability [18,19,20,21]. These polypeptides accumulate in seeds during the late maturation stage, i.e., they are present in dry seeds and are not anymore detectable after germination [19]. The promoters of the Arabidopsis *LEA* genes contain cis-elements sensitive to abscisic acid (ABA), drought and low temperature [18]. Chen et al. reported an increase in the potato *LEA* gene expression in response to drought, salinity, extremely high and low temperatures [22].

LEA-proteins are characterized by high glycine (Gly) contents, low amount (or even lack) of cysteine (Cys) and tryptophan (Trp) residues, and predominance of alanine (Ala), glutamate (Glu), lysine/arginine (Lys/Arg) and threonine (Thr) [23]. Due to these primary structure features, LEA proteins are stable in a broad temperature range and are highly hydrated. During cell dehydration, LEA-proteins act as chaperons, i.e., impact on structural stabilization of other proteins and cell membranes by intensive hydrogen bond formation, they stabilize denatured proteins and promote their refolding [19]. LEA proteins were shown to sequester ionic compounds, accumulating during cell dehydration, and to protect membrane proteins and enzymes from the deleterious effects of increased salt concentrations [19]. Although the LEA proteins were detected in all cell compartments, they are predominantly localized in cytoplasm [24]. The most of the LEA-proteins adopt unordered, randomly coiled structure in aqueous solutions [19]. However, dehydration results in their re-folding to yield the structures with higher impact of amphiphylic α-helix. Flexible structural elements (such as polyproline II helices), which enhance binding to DNA, RNA or other proteins, are also characteristic for LEA proteins [18,19].

Altogether, 51 genes encoding LEA proteins were identified in Arabidopsis genome [18]. According to the Pfam protein domain database, they can be distributed in eight families: Dehydrins (PF00257), LEA-1 (PF03760), LEA-2 (PF03168), LEA-3 (PF03242), LEA-4 (PF02987), LEA-5 (PF00477), LEA-6 (PF10714) and seed maturation protein (SMP, PF04927) [18,20]. LEA proteins, as well as some heat shock proteins, belong to the group of so-called moonlighting proteins, which are characterized with several physiologically relevant functions [20,25]. The expression levels of *LEA* genes are much higher in seeds than in vegetative organs of plants. Indeed, in total, 21 of 51 Arabidopsis *LEA* genes are expressed only in seeds and are mostly directly involved in the control of their formation [18]. This tendency is even more pronounced in *Camellia sinensis*: 39 among 48 *LEA* genes detected in this plant might play an important role in seed maturation [26].

The largest group of LEA-proteins is represented by the LEA-2 family [18,20,26]. The proteins representing this family are featured with higher content of hydrophobic amino acids and conservative three-dimensional structure. *Dehydrins* represent the most studied group of LEA proteins, and comprise the family of universal protective molecules involved in plant responses to various abiotic stressors. Binding and structural stabilization of cellular biopolymers by dehydrins relies on their homo-and hetero-complexes, which are often multimeric [27]. In agreement with the fact, that decrease in cellular levels of dehydrins can enhance sensitivity of plant tissues to desiccation [2], the orthodox, intermediate and recalcitrant seeds differ in dehydrin composition and contents [28].

### 2.2. Heat Shock Proteins (HSPs)

Activation of the HSP system represents one of the most universal responses of living organisms to stressors. Based on their molecular weights, eukaryotic HSPs can be distributed in five classes: Hsp100, Hsp90, Hsp70, Hsp60, Hsp40 and small HSPs (sHSP).

Among the whole HSP superfamily, sHSPs represent the largest and the most heterogeneous (in comparison to the other four) group of proteins with molecular weights varying in the range of 12–43 kDa and are characterized with a highly conservative C-terminal α-crystalline domain containing 80–100 amino acid residues [29]. Similarly to LEA proteins, sHSPs are accumulated at the late seed maturation stage and are present in their native state in dry seeds [8,29,30]. In seeds, sHSP promote folding of newly synthesized proteins, antioxidant protection, as well as refolding of polypeptides with damaged tertiary structure [8,29,30,31]. One of the most important features of sHSPs is their ability to form large oligomeric complexes (100–1000 kDa), the size of which can reach 5000 kDa under stress conditions [29]. Apparently, only relatively large oligomers have high chaperone activity, i.e., able to interact with damaged or misfolded proteins and stabilize their structure [29].

The Arabidopsis family of heat stress transcription factors (HSFs) comprises 21 members in three classes (A, B, and C) [32]. Kotak et al. analyzed the performance of HSFA9 which is expressed in Arabidopsis seed exclusively at the late maturation stage [33]. The authors demonstrated that the expression of HSFA9 was regulated by the seed-specific transcription factor ABI3. Indeed, the ABI3 knockout lines lacked detectable levels of HSFA9 transcripts and proteins. On the other hand, ectopic expression of *ABI3* triggered accumulation of HSFA9 in transgenic plantlets in response to application of ABA. Furthermore, it was shown that ABI3 could activate the *HSFA9* promoter, whereas HSFA9, in turn, proved to be a potent activator of some *HSP* genes [33]. In addition, analogously to the *LEA* genes, expression of *HSPs* can be mediated by the key dormancy regulator DELAY OF GERMINATION (DOG1) [34]. Moreover, expression of several seed *HSP* genes was shown to be induced by osmotic stress. For example, dehydration triggers expression of *HaHSP17.6* and *HaHSP17.9* genes in the seeds of sunflower (*Helianthus annuus*), and the levels of corresponding mRNAs correlated with the degree of the water loss [29]. The embryogenesis of soybean was reported to be accompanied with accumulation of specific *HSP* transcripts, and the levels of individual mRNAs positively correlated with seed longevity [14]. It was shown recently, that expression of 41 *RcsHSP* genes encoding cytoplasmic, mitochondrial and microsomal α-crystalline domain-containing HSPs, accompanied maturation of castor bean (*Ricinus communis)* seeds [35]. Kaur et al. reported an essential increase in the abundance of the *OsHSP18.2* transcripts in rice seeds at the late maturation stage [31]. Thereby, the expression levels significantly increased after accelerated aging, but dramatically decreased after beginning of germination. It was shown further, that expression of *OsHSP18.2* improved seed vigor and longevity by reducing accumulation of deleterious ROS in stored seeds [31].

### 2.3. Non-Reducing Carbohydrates

Accumulation of non-reducing carbohydrates (predominantly sucrose, raffinose and stachyose), which stabilize the structure of membranes and cytoplasmic proteins by replacing water molecules, is one of the most important strategies of seed survival during desiccation. In contrast, the presence of reducing monosaccharides negatively correlates with seed longevity [8]. It can be explained by enhanced glycation of cellular proteins with reducing carbohydrates [36], their phosphorylated derivatives [37] and carbonyl products of sugar degradation, which are mostly represented by α-dicarbonyl compounds (3-deoxyglucosone, glyoxal, and methylglyoxal) [38]. This process is known, to be affected by environmental stressors and ageing [39,40].

The patterns of soluble sugars change essentially at the late seed maturation stage: the abundance of hexoses (glucose and fructose, as well as their phosphorylated derivatives) gradually decreases, whereas non-reducing oligosaccharides accumulate [8,41]. During dehydration, oligosaccharides and LEA proteins replace water as a hydrogen-bonding partner of the phosphate groups in polar heads of phospholipids [42]. This results in transition of the liquid-crystalline state of membranes to the gel phase, which is accompanied with a decrease in lipid mobility due to stronger van der Waals interactions between lipid heads after the loss of their hydrate shells [42,43]. The cytoplasm of dehydrated cells becomes “glassy” without the transition of cytoplasm to the solid state. Thereby, the viscosity of cytoplasm increases, whereas diffusion of water and oxygen get suppressed and the rates of all possible chemical reactions become dramatically reduced [5,42,43]. Due to these shifts in the physicochemical properties of multiple cellular structures, orthodox seeds are able to maintain viability for decades [30]. 

## 3. Epigenetic Regulation of Desiccation Tolerance

Epigenetics is the field of biology, specifically addressing heritable (both via mitosis- and meiosis-related mechanisms) alterations in genome, which are not accompanied with any changes in DNA sequence [44,45]. Such epigenetic events mainly rely on DNA methylation at cytosine residues, post-translational covalent modifications of histones (e.g., acetylation, methylation and ubiquitylation), and synthesis of small RNAs [44,45].

DNA methylation is involved in both negative and positive regulation of gene expression, representing thereby one of the most well-characterized epigenetic regulatory mechanisms in plant development [46]. It ensures genome stability by inactivating potentially dangerous elements—transposons and foreign DNA sequences [47]. On the other hand, plants use DNA methylation to maintain genome plasticity, which allows efficient adaptation to changing environmental conditions [46,48]. This modification underlies genomic (parental) imprinting, cell differentiation, embryo formation, as well as seed maturation and germination [46,49,50]. It is assumed, that DNA methylation also plays an important role in the formation of desiccation tolerance at the late seed maturation stage [51,52].

DNA methylation is an ubiquitous covalent modification of cytosines in DNA sequence and can be of either maintenance or de novo type [48,53]. In all eukaryotic organisms, the modification sites are typically localized in the CG consensus, although in plants, methylation can also occur at the cytosines localized in the CHG and CHH consensus sequences, where H is any nucleotide excepting G [46]. The methylation reaction is catalyzed by site-specific DNA cytosine methyltransferases and yields 5-methylcytosine residues in the DNA chain [46]. In the case of de novo methylation, the substrate is a non-methylated DNA molecule. Maintenance methylation assumes modification of newly synthesized DNA strands, complementary to the parent DNA, to preserve the existing patterns of cytosine methylation. 

Plants have at least three families of DNA methyltransferases, which are represented by (i) methyltransferases 1 (MET1), (ii) chromomethylase 3 (CMT3) and (iii) domain rearranged methyltransferases 2 (DRM2). MET1 is involved in maintenance methylation at CG sites, whereas CMT3 catalyzes maintenance methylation at CHG and CHH sites [46]. The methyltransferase DRM2, which catalyzes de novo methylation, is expressed in all organs and tissues of the plant organism. The recognition of target methylation sites relies on small interfering RNAs complementary to the target DNA loci. After recognition and interaction with the corresponding loci, the RNA-directed DNA methylation (RdDM) occurs at CG and, to a less extent, at CHG and CHH sites [53]. Methylation of CHH sites via the RdDM mechanism was demonstrated during seed formation and at the late maturation stages [50]. 

During seed formation, DNA methylation at the CG and CHG sites remain, in general, stable, whereas the levels of mCHH methylation noticeably increase throughout the whole process of seed development, and later on gradually decrease during seed germination [50,53]. The genome regions, hypomethylated during the whole plant life cycle, are essentially enriched in genes encoding transcription factors, storage proteins and enzymes of fatty acid metabolism [54]. Mature embryos are characterized by a higher level of genome methylation compared to the embryos at the early stages of development and seedlings, mainly due to the high level of methylation at the CHH sites.

An et al. studied DNA methylation in the developing seeds of soybean (*Glycine max*) and identified 40, 66, and 2136 genes containing differentially methylated regions at the CG, CHG, and CHH sites, respectively [55]. Methylation was detected in 66, 45 and 9% of the CG, CHG and CHH sites, respectively. Thereby, the CHH methylation levels increased during seed maturation from 6% to 11%, whereas the expression of the genes, containing the CHH consensus, was mostly reduced. These genes were predominantly associated with DNA replication and cell division [55]. 

Seed desiccation at the late maturation stage also initiates methylation of nuclear DNA [51,56], which is often associated with long-term repression of gene transcription [46]. Michalak et al. addressed the effect of desiccation on the orthodox seeds of wild pear (*Pyrus communis* L.) [56]. The authors found an increase in the overall DNA methylation levels immediately after completion of seed maturation [56]. Remarkably, this effect was detectable during two years afterwards. However, recalcitrant maple seeds (*Acer pseudoplatanus*), which were dried in the presence of silica gel from 47.7 to 13.9–35.0%, showed decreased 5′-methylcytosine contents in the embryonic axes and cotyledons by 27 and 37%, respectively [51]. Apparently, an increase in the levels of DNA methylation during desiccation is characteristic only for orthodox seeds.

Despite the cytosine methylation is a relatively stable epigenetic modification, it is dynamically controlled by enzymatic demethylation [57]. Demethylation is accomplished by the excision of methylated nucleotides and subsequent insertion of non-methylated cytosines [58]. The following enzymes are involved in demethylation of plant DNA: demeter (DME), repressor of silencing 1 (ROS1)/demeter-like 1 (DML1), DML2 and DML3 [59]. The DME protein is required for the imprinting of the paternal or maternal alleles of certain genes, while ROS1, DML2 and DML3 are active in vegetative tissues. ROS1 mediates RdDM-dependent and RdDM-independent methylation and can prevent the spread of DNA methylation from transposable elements to protein-coding genes. 

It is important to note, that besides DNA methylation, epigenetic regulation of gene expression can also rely on covalent post-translational modifications (PTMs) of histones and chromatin remodeling [48]. Such PTMs of histones as acetylation, methylation, and ubiquitination play an important role in the regulation of seed formation and dormancy [46,60,61]. Small interfering RNAs and/or long non-coding RNAs can also trigger epigenetic changes in dormant or germinating seeds. For example, Huo et al. found that the regulatory effects of DOG1 on seed dormancy, at least partly, are mediated by miRNA regulated pathways [62]. Thus, DOG1 can affect seed dormancy and flowering in lettuce (*Lactuca sativa* L) and Arabidopsis via alteration of miR156 and miR172 levels. Suppression of *LsDOG1* enabled seed germination at elevated temperatures and promoted early lettuce flowering in association with reduced miR156 and increased miR172 levels. In Arabidopsis, higher miR156 levels (due to over-expression of the MIR156 gene) enhanced seed dormancy and delayed flowering [62].

## 4. Seed Longevity in the Context of Dormancy and after-Ripening

### 4.1. Seed Longevity

Seed longevity (also referred to as storability and seed life span) is one of the principal target parameters in modern agriculture, which is especially important in the context of global climate change and is recognized as critical for plant biodiversity conservation [7,63,64]. Seed longevity is usually defined as the maximal possible time (calculated from the completion of seed maturation till the initiation of its germination) when seeds still preserve viability [1]. The longevity of seeds depends on their structure and chemical composition, as well as on the environmental conditions of their development [7,63,65]. Thus, during the late maturation stage, seed longevity is influenced by the mother plant [64]. In general, higher longevity of seeds can be considered as one of the adaptive mechanisms, which allow plant populations spreading in time and space [8]. In different plant species longevity varies from several years to decades. However, seeds of some species can preserve their viability during hundreds and even thousands years [63]. The palm *Phoenix dactylifera* L., which seeds, dated by Christos times, were found in the fortress of Herodian, can serve as an example of such plants [66].

In the beginning of this decade, Nguyen et al. identified five loci of quantitative traits of seed longevity (*Germination Ability After Storage, GAAS1-5*) in Arabidopsis inbred lines [67]. The *GAAS* loci were shown to be co-localized with seed dormancy locus *DOG*. The detailed expression analysis of the quantitative trait loci *GAAS5* and *DOG1* revealed a correlation of deep seed dormancy with low seed longevity and vice versa. However, Dekker et al. showed that the seeds of *dog1-1* mutants exhibit a reduced lifespan and lower levels of *LEA* and *sHSP* expression [34]. The mechanisms responsible for seed longevity are likely to correlate with the mechanisms underlying regulation of dormancy, but the nature of such interaction is still unknown.

### 4.2. Seed Dormancy

Seed dormancy is typically referred to as the physiological state of normally developed viable seeds with a temporal germination block, duration of which is independent from environmental conditions [68,69,70]. The main factors involved in the regulation of onset and termination of dormancy, are DOG proteins and the ratio of ABA and gibberellins (GAs), specifically GA1, GA3 and GA4 [60,71,72]. Seed dormancy can rely on different mechanisms and may be associated with incomplete development of embryo morphology (morphological dormancy), hardening of seed coat, which prevents imbibition (physical dormancy) and/or low GA level (physiological dormancy) [69,70]. Physiological dormancy can be terminated by incubation of imbibition seeds at decreased (0–10 °C) or increased (>15 °C) temperatures, or by treatment of seeds with GAs, karrikins, nitrates, or inhibitors of ABA biosynthesis [72]. This results in the shift of ABA/GA balance towards the higher levels of the latter. This, in turn, leads to the activation of GA-related signaling pathways involved in control of seed germination. In the natural way, the termination of seed dormancy triggered by so-called after-ripening (AR), i.e., storage under dry conditions [72] (Figure 1B). 

### 4.3. Seed After-Ripening

Seed after-ripening (AR) is a complex process, which still remains to a great extent under-explored. It is triggered by separation of the seed from the mother plant and is accompanied by a gradual increase of its germination potential [72,73,74]. AR is characterized by a decrease in ABA levels and reduced seed sensitivity to this hormone in parallel to the increase in GA levels [75,76]. This process is enhanced by improvement of tissue oxygen supply and increase of storage temperature, whereas even a minimal increase in seed water contents suppresses AR [77,78]. After completion of this developmental phase, seeds become able to germinate under a broad range of temperature and irradiation conditions [79]. It is important to note, that the depth of seed dormancy (i.e., the time required for its termination) is defined not only genetically, but also by the environmental conditions of seed development and maturation (temperature, nutrient and water supply) [80]. 

Already at the end of the last decade, Carrera et al. identified the main groups of *Arabidopsis* genes, demonstrating differential expression patterns upon dry storage of seeds [75]. The authors report the core gene sets, positively or negatively regulated by dry storage. Each set included the genes encoding repression or activation of ABA signaling: *LIPID PHOSPHATE PHOSPHATASE 2* (*LPP2*) and *ABA DEFICIENT1* (*ABA1*), respectively. The set of the genes, down-regulated by AR, included *ABA1* involved in ABA synthesis. On the other hand, the set, up-regulated under AR conditions comprised LPP2. Recently, Ishikawa et al. identified 1730 phosphopeptides in a large-scale liquid chromatography-mass spectrometry (LC-MS)-based analysis of ABA-related phosphoproteome of barley seed embryos [76]. Analysis of phosphoproteome of the embryos isolated from the freshly harvested seeds and those after AR, showed significant differences in the patterns of ABA-responsive phosphosites. The obtained results were supported by peptide motif analysis, which indicated activation of a new set of protein kinases during after-ripening.

Unfortunately, to date, it remains unclear how these complex regulatory events can occur in dehydrated seed tissues. One possible explanation could be differential distribution of water between seed structures and individual biomolecules within them. The assessment of proton mobility in tobacco seeds by nuclear magnetic resonance (NMR) spectroscopy imaging, accomplished by Metzger, supported this hypothesis, i.e., demonstrated uneven distribution of water molecules in dry seeds [74]. The author showed, that higher levels of proton mobility indicating higher contents of water and/or oils, is characteristic for the micropillar endosperm layer. Indeed, the expression of *β*-1,3-glucanase- the enzyme involved in hydrolysis of cell wall *β*-glucans to oligosaccharides, was localized in this zone [74]. Recently, reactive oxygen species (ROS) were proposed as regulators of seed maturation [79]. Thus, overproduction of ROS can result in site-specific oxidation of mRNA molecules, along with oxidation and carbonylation of proteins, associated with desiccation/re-hydration-related signaling pathways [80]. 

In general, the physiological state of seeds during dormancy, AR and prolonged storage is defined by the contents of water in their tissues. Thus, for successful AR, dehydrated state of seeds is critical: even a small and transient increase of water contents can interrupt dormancy and trigger seed damage. Therefore, understanding of the mechanisms behind the survival of seeds after desiccation is one of the central problems of modern seed physiology. 

## 5. Role of ABA and DOG1 in the Regulation of Seed Maturation

ABA is involved in regulation of several key events, accompanying seed maturation: biosynthesis and transport of nutrients, degradation of chlorophylls, dehydration of seed tissues and onset of dormancy [13]. Additionally, this hormone impacts on suppression of the genes encoding germination-related proteins: α-amylases, lipase, lipid transfer proteins [8,81,82]. 

At the early steps of embryogenesis, the developing embryo is controlled by ABA transported from the mother plant tissues, but at the later developmental stages, the hormone is produced by the seed itself [71,83,84]. In the beginning of embryogenesis, ABA prevents seed abortion and promotes embryo growth. At the final stages of embryogenesis, in contrast, the levels of ABA increase. This results in the suppression of embryo growth, i.e., in this case, ABA acts as a GA antagonist [83,84]. Once the embryo has been formed, its size begins to increase by cell elongation and due to accumulation of storage compounds. At the cellular level, ABA blocks transition of the embryonic cells from the G_1_- to the S-phase of cell cycle, stimulates transport of monosaccharides, amino acids from mother plant and triggers the synthesis of their storage forms—polysaccharides and proteins, respectively. At the late maturation stage, metabolic processes are gradually slowed down, the seeds get dehydrated and enter dormancy [68,81]. The regulation of seed maturation mostly relies on the network of transcription factors known as LAFL (LEAFY COTYLEDONS 1 (LEC1), ABA-INSENSITIVE 3 (ABI3), FUSCA 3 (FUS3) and LEAFY COTYLEDONS 2 (LEC2)) [61], as well as DOG1 and DOG4, which are associated with ABA-dependent seed maturation regulators [34,60,85]. These factors control expression of the seed genes involved in regulation of embryogenesis, suppression of germination, accumulation of storage compounds and onset of desiccation tolerance. The mutants *abi3* and *fus3* are sensitive to dehydration, and the seeds of *fus3* germinate directly on the mother plant [8,71,86,87]. LEC2, FUS3 and ABI3 also regulate expression of the genes encoding the storage proteins—oleosins and globulins. The seeds of *abi3*, *fus3* and *lec2* mutants contain lower amounts of storage proteins and lipids [8,81,82,86]. The transcription factor DELAY OF GERMINATION 1-like 4 (DOGL4), which triggers expression of about 70 genes specific for seed maturation, including those encoding such storage proteins as albumin, cruciferin and oleosin, is believed to be the main regulator of storage compound accumulation in seeds [85]. In addition, the expression of *DOGL4* gene is induced by ABA, while the expression of *DOG1* is independent from ABA [85].

ABA plays a crucial role in the late stages of seed maturation [83]. This hormone was shown to activate the synthesis of LEA-proteins, which impact on desiccation tolerance of developing seeds and on their tolerance to adverse environment factors [8,82]. Thereby, the transcription factors ABI3 and ABI5 represent the most important players, involved in the control of seed maturation, metabolism of raffinose family oligosaccharides and expression of *LEA* genes [34,61,88]. It was shown that ABA-insensitive seeds of Arabidopsis *abi3* and maize *vp1* mutants are unable to accumulate storage proteins, do not acquire desiccation tolerance, do not enter dormancy and might germinate directly on the mother plant (vivipary) [81]. Moreover, the transcription factors ABI3 and ABI5 are proposed to be involved in regulation of the chlorophyll degradation machinery, activated at the end of seed maturation [8,14,88,89]. 

Chlorophylls are synthesized during the early embryogenesis, participate in the photochemical photosynthetic reactions and degrade at the late maturation stage [90,91,92]. However, this degradation often remains incomplete [93]. For example, mature seeds of the pea (*Pisum sativum* L.) and alfalfa (*Medicago truncatula* L.) *abi5* mutants retain significant amounts of chlorophylls [88]. As ABI5 controls not only the synthesis of LEA-proteins and raffinose family oligosaccharides, but is also involved in regulation of chlorophyll degradation enzymes, Zinsmeister et al. consider this factor as the most important regulator, affecting the maintenance of seed viability during dormancy [88]. Recently, new major regulators of desiccation tolerance were identified in Arabidopsis seeds in addition to the LAFL network: plant AT-rich sequence- and zinc-binding proteins 1 and 2 (PLATZ 1 and 2), and AGAMOUS-like 67 (AGL67) polypeptide [94]. González-Morales et al. showed that over-expression of PLATZ1 and AGL67 or dehydration-responsive element-binding protein 2D (DREB2D) at least partially compensates the loss of desiccation tolerance in mutant *abi3-5* [94]. The authors assume that the signal cascade controlling seed desiccation tolerance includes the transcription factors PLATZ1, PLATZ2 and AGL67, which play after LEC1, ABI3, FUS3 and LEC2. 

The completion of embryogenesis and onset of dormancy are controlled by the transcription factors LEC and FUS3, i.e., by the signaling pathways related to phytohormones—ABA, GA and ethylene [68,95,96]. Indeed, ABA was found to stimulate LEC1- and FUS3-mediated protein synthesis. Furthermore, FUS3 triggers up-regulation of ABA tissue levels, providing a positive feedback for ABA- and FUS3-controlled events. LEC1 was shown to activate LEC2 and FUS3, while LEC2 activates LEC1 and FUS3 [71,81,95]. The expression of the genes, encoding GA 3-oxidases 1 and 2 (*GA3ox1* and *GA3ox2,* respectively), i.e., the enzymes involved in conversion of GA precursors into their biologically active forms, is suppressed by FUS3 and LEC2. Besides, LEC2 induces expression of the factor AGL15, which, in turn, activates expression of GA 2-beta-dioxygenase 6 (*GA2ox6)* gene. Recently, Braybrook et al. showed that GA is implicated in the repression of *LEC* genes in seedlings, i.e., this regulatory pathway is not suppressed by germination [95]. 

**Figure 2:**
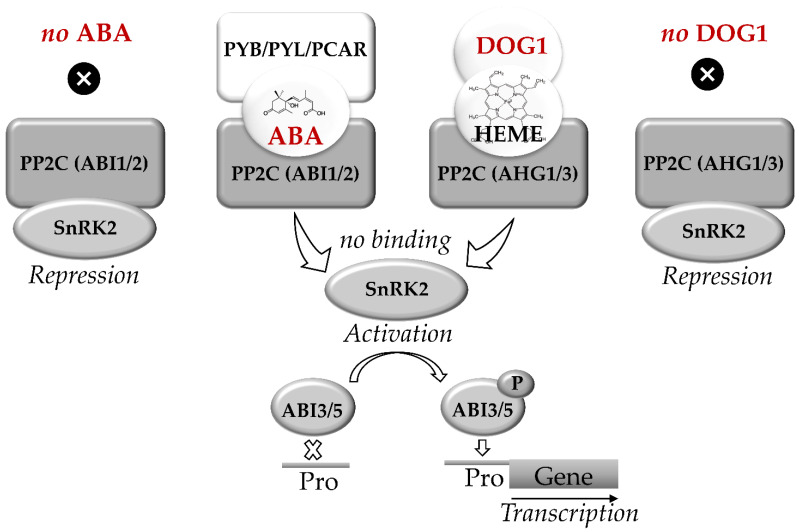
Abscisic acid (ABA) and DELAY OF GERMINATION 1 (DOG1) signaling pathways in seeds. C encoded by ABI1 and ABI2 genes. The key elements of DOG1 signaling are heme molecule and PP2Cs encoded by *AHG1* and *AHG3* genes. Triplex complexes of PCAR-ABA-PP2C and/or DOG1-HEME-PP2C block the binding of PP2C to SnRK2. The active SnRK2 phosphorylates ABI3 and ABI5 which bind to the promoters (Pro) of ABA-controlled genes. In seeds, the parallel ABA and DOG1 signaling pathways activate synthesis of RFO, expression of *LEA* and *HSP* genes, thus regulating the onset of desiccation tolerance and transit to dormancy. Based on [34,60,82,87,97,98].

Recently regulation of seed dormancy via the interaction of ABA- and DOG1-related signaling pathways was reported and comprehensively discussed in literature [60,87,97,98]. Nakabayashi et al. proposed that DOG1 and ABA act in mostly parallel pathways involved in regulation of dormancy [99]. These pathways merge downstream at ABI3 or ABI3. The key elements of ABA signaling are represented by the receptor Pyrabactin resistance/PYR-like/Regulatory component of abscisic acid receptor (PYR/PYL/RCAR) and protein phosphatases type 2C (PP2C), encoded by *ABI1* and *ABI2* genes (Figure 2). PP2C are the negative regulators of sucrose-non-fermenting-related kinases (SnRK2), which phosphorylate the transcription factors ABI3 and ABI5 [71,82,97].

Group A PP2C proteins, which are known as the key negative regulators of ABA signaling are represented by two subfamilies, namely ABI1 and ABA Hypersensitive Germination1 (AHG1) [98]. At low ABA levels, PP2C blocks transduction of the ABA signal by suppressing SnRK2, blocking thereby the regulatory events mediated by ABI5 and ABI3. In contrast, the increase in ABA levels triggers binding of PP2C to the receptor, forming a triple ABA-RCAR-PP2C complex. This complex can block the activity of PP2C phosphatase. This, in turn, triggers phosphorylation of the transcription factors ABI5 and ABI3 and induces the expression of ABA-dependent genes [96,97].

DOG1 was demonstrated to trigger ABI5/ABI3-mediated expression of *LEA* and *HSP*. This might enhance accumulation of nitrogen-containing compounds and promote completion of seed maturation and subsequent desiccation [34]. The key elements of DOG1 signaling are represented by the protein phosphatases ABA Hypersensitive Germination 1 and 3 (AHG1 and AHG3y), which are the members of the PP2C family and also suppress protein kinases SnRK2 [98]. Furthermore, DOG1 was shown to bind heme. Binding of DOG1 to AHG1 and to heme represents two independent events, both of which are essential for DOG1 function. The binding of DOG1 to AHG1 and/or AHG3 results in release of SnRK2 and phosphorylation of ABI5. Moreover, DOG1 was shown to affect the expression of multiple genes controlling seed maturation, including *LEA* and *HSP*, as well as the genes of RFO biosynthesis. This function of DOG1 protein is partially complemented the transcription factor ABI5 [34].

Thus, activation of the signaling pathways regulated by ABA and DOG1 results in the inhibition of PP2C family protein phosphatases, which suppresses the transcription factors responsible for the expression of ABA-dependent genes [87].

## 6. Mechanisms of Seed Antioxidant and Redox Protection

### 6.1. Seed Damages

After completion of AR and termination of dormancy, seeds become ready for germination [72]. However, for meeting the optimal environmental conditions for germination, seeds need to preserve viability for long times (months, years, or even decades) [60,68]. Such a prolonged viability is primarily caused by the lack of free water in seeds and non-specific inhibition of all enzymatic activities. Nevertheless, long-term storage, as well as combination of increased humidity and ambient temperature (i.e., higher than the storage optimum) of the environment, can promote deleterious alterations in seed metabolism and cause structural changes in seed proteins, which are often referred to as seed damage. This damage is typically manifested by compromised functions of individual polypeptides, at the molecular level caused by non-enzymatic modifications of proteins with ROS and carbonyl compounds accompanied with accumulation of oxidation [100,101] and advanced glycoxidation end products (AGEs) [102]. Surprisingly, glycation of seed proteins was addressed only in few studies till now [36,103]. As glycation was recently proposed to be involved in plant signaling [104], this aspect needs to be elaborated in more detail. Nevertheless, numerous studies proved that seed damage during storage is caused by accumulation of ROS, which can react with almost all biological molecules, including lipids, DNA, and proteins [52,79]. Overproduction of ROS (which represent, to a large extent, free radicals) result in enhancement of lipid peroxidation, which, along with AGE formation, might lead to destruction of cell membranes [64,102,105,106].

### 6.2. Seed Redox Homeostasis and Antioxidant Protection 

The metabolism of all aerobic organisms, including plants, is based on an array of redox processes, critically important for both catabolic and anabolic pathways [107,108,109]. Redox reactions in electron transport chains (ETCs) of mitochondria and chloroplasts are characterized with high rates of electron or/and energy transfer, that inevitably lead to the formation of reactive oxygen species (ROS). The formation of ROS can rely on electron or energy leakage to O_2_ [110], as well as on the enzymatic reactions catalyzed by NADPH oxidases, glycolate oxidases, amine oxidases, and cell-wall-bound peroxidases [111,112,113,114]. In parallel to ROS, reactive nitrogen, sulfur and carbonyl species (RNS, RSS and RCS, respectively) [102,103,115,116,117] can form in seeds, although only limited information about the physiological role of RSS and RCS is available [117].

ROS are the products of constitutive plant redox metabolism and are, therefore, continuously generated in plant tissues, being involved in multiple normally occurring in cell physiological processes [118,119,120]. Thus, ROS generation is one of the earliest responses of plant cells to various biotic and abiotic stresses, as well as internal regulatory events [121]. In the most of the cases, superoxide anion radical (O_2_^∙^) is produced as a primary ROS type. This intermediate can be easily converted to hydrogen peroxide (H_2_O_2_) and other peroxides, which are, in turn, readily involved in Fenton reaction, yielding hydroxyl radical (^∙^OH) [109,122]. Other chemically active oxygen-containing species are also referred to as ROS, in particular—triplet chlorophyll (^3^Chl) and singlet oxygen (^1^O_2_) [52,105,111].

Being by-products of electron transport in ETCs [123,124], ROS are acting as signals in distinct biological processes, such as growth and development, responses to biotic and abiotic stresses, programmed cell death [111,119], where they are typically act as secondary messengers in a broad array of signaling networks [114,125,126]. During seed formation, generation of ROS accompanies several developmental steps—maturation, desiccation, dormancy, aging, and germination [111,118,127]. In particular, ROS are involved in the regulation of embryogenesis and seed germination [105,111]. ROS are involved in the control of dormancy release by dry storage of seeds (during after-ripening). They mediate perception of environmental triggers of germination and are involved in signal transduction. ROS are playing the signaling role and act as secondary messengers only when the ROS contents are within the so-called “oxidative window” [111,118,120]. The basal level of ROS in cells is essential for supporting cell proliferation and differentiation. Moreover, cell death (which was previously thought to be the outcome of oxidative stress) is now considered to be the result of programmed events triggered by ROS [120]. 

Under normal conditions, continuous generation of ROS is accompanied with their detoxification by a broad range of low-molecular weight and enzyme-based radical scavenging and antioxidant systems [128]. However, when production of ROS overwhelms the activity of these antioxidant protective systems, their tissue contents increase and oxidative stress develops [112,129]. At the molecular level, oxidative stress is manifested by enhanced lipid peroxidation, disruption of membrane integrity, inactivation of enzymes, oxidative degradation of proteins and of nucleic acids along with depletion of the antioxidant pools. Since the hydroxyl radical (^∙^OH^·^) is short-living, its detoxification by enzymatic mechanisms seems to be impossible [105]. To date, no specific scavengers of OH^·^ are known. The effects of unspecific scavengers (mannitol, sorbitol, dimethyl sulfoxide, thiourea) are mainly dependent on the nature of corresponding ROS precursors of the hydroxyl radical (hydroperoxyl, peroxynitrite, H_2_O_2_ or superoxide), although the presence of transition metals in solutions and efficiency of their chelation are also important [109]. 

In contrast to hydroxyl radical, detoxification O_2_^∙^^−^, H_2_O_2_ and ^1^O_2_ can be mediated by antioxidant enzymes [109]. Thereby, superoxide dismutases (Cu-SOD, Zn-SOD, Mn-SOD, Fe-SOD), peroxidases (guaiacol peroxidase, ascorbate peroxidase, glutathione peroxidase), catalase play the crucial role in antioxidant protection of seeds. Additionally, low molecular weight antioxidants—tocochromanols (i.e., tocopherols and tocotrienols), carotenoids, ascorbate and glutathione, can be involved [16,17,52]. 

The ROS levels in seeds are also controlled by the enzymes of the ascorbate-glutathione cycle also known as Foyer-Halliwell-Asada pathway [130,131]. The reduced form of ascorbate is able to directly interact with ROS, as well as participate in the reduction of other low molecular weight antioxidants—tocopherols, glutathione. Tocopherols are universal protectors of cell membranes, participating in the quenching of ROS and thus preventing non-enzymatic oxidation of lipids [16,105]. Glutathione can directly act as a ROS-quenching molecule or can serve as an electron donor for enzymes involved in ROS detoxification, for example, glutathione S-transferase [132]. The interaction of glutathione with ROS is accompanied by oxidation of its sulfhydryl group and conversion in glutathione disulfide. Glutathione reductase, which reduces oxidized glutathione to its sulfhydryl form, is present in dry seeds and is rapidly activated upon hydration [52,130]. The ratio of the reduced and oxidized forms of glutathione is also considered as a marker of seed viability [15,65,132,133]. In addition, tocochromanols can essentially affect the glutathione contents [132,134].

Reactive nitrogen species (RNS), in particular nitric oxide (NO), are the biological messengers, which orchestrate a plethora of plant functions, mainly via non-enzymatic post-translational modifications (PTMs), such as S-nitrosylation or tyrosine nitration. Hundreds of proteins have been identified as potential targets of NO-related PTMs [135]. This illustrates the importance of NO as a key signaling molecule tremendously impacting on plant growth, development and senescence [119,136,137]. Indeed, the NO-signaling pathway is involved in regulation of seed germination, growth of pollen tube, root organogenesis, flowering and fruit ripening [138,139,140]. Similarly to ROS, RNS play an important role in mediating and regulation of plant response to abiotic stress and in plant interactions with ecosystem partners [140,141]. As was shown in the last decade, the exogenous NO can interrupt seed dormancy and promote seed germination [142,143,144]. These effects clearly indicate that NO is involved in modulation of ABA-related signaling pathways, affecting seed dormancy and germination [145,146].

## 7. Repair of Nucleic Acids and Proteins in Germinating Seeds

Under the appropriate environmental conditions (i.e., humidity, temperature and irradiation optimal for each species) metabolic processes in seeds are re-activated and germination is initiated. Successful germination of mature seeds essentially depends on the efficiency of damage repair systems. Damage is inevitably accumulated in DNA, RNA, and proteins during sequential periods of dehydration and rehydration [147]. The repair is triggered by tissue re-hydration, when all involved enzymatic systems are reactivated and continuous energy supply is secured by enhancement of aerobic respiration in mitochondria [148,149]. The expression of the genes encoding enzymes involved in the repair of nucleic acids and proteins is strongly up-regulated at the late stages of seed maturation. This ensures fast translation of required enzymes during the imbibition stage [8,30,148].

The repair of damaged DNA in seeds relies on the universal mechanisms, typical for all eukaryotic cells: repair of double-strand breaks by homologous recombination and non-homologous end-joining, nucleotide excision repair, base excision repair and correction of unpaired DNA bases [150]. Oxidative damage of seed DNA is mostly associated with either desaturation of deoxyribose or covalent modifications of bases [30]. As was shown for Arabidopsis, hydroxylation of guanine yields a potentially mutagenic base 7,8-dihydro-8-oxoguanine (8-oxoG) as the major oxidative modification of seed DNA [151]. This base can form complementary pairs not only with cytosine, but also with adenine, that might result in errors during replication [148,152]. The repair, i.e., removal of damaged bases (in particular, 8-oxoG) from the DNA double helix, typically relies on the base excision repair (BER) system [151]. First, the damaged base is recognized and removed by cleavage of the corresponding N-glycosidic bond by DNA glycosylase [152]. The resulted apurinic/apyrimidinic (AP) sites are the substrates of the 8-oxoguanine DNA glycosylase/lyase, which cleaves the sugar-phosphate backbone of DNA to form a single-strand break, before DNA polymerase beta (POLB) the chain repair [151]. Thus, BER includes the excision of the damaged DNA base, cleavage of the sugar-phosphate backbone at AP sites, clean-up of the resulting DNA ends, filling of gaps by DNA synthesis, and DNA ligation [152].

The other types of DNA damage, like double-strand breaks, also require specific repair machinery [148]. These base damages can be repaired by DNA glycosylases—8-oxoguanine DNA glycosylase/lyase (OGG) and formamidopyrimidine-DNA glycosylase (FPG) [151,153]. After the subsequent cleavage of the sugar-phosphate backbone, the breaks can be restored by DNA ligases [64,148]. The critical role in the repair of dehydration-related DNA damage plays poly (ADP-ribose) polymerase (PARP), which is catalyzing poly-ADP-ribosylation—reversible covalent modification of proteins by an ADP-ribose homopolymer [154]. The catalytic activity of PARPs is stimulated by DNA strand breaks. PARP is also involved in transcriptional regulation, formation of mitotic spindle during cell division, intracellular trafficking etc. In plants, activation of PARP superfamily members is the marker of stress responses [154,155,156,157]. This enzyme is featured with two catalytic functions: NAD^+^-hydrolase and ADP-transferase. The hydrolysis of NAD^+^ yields ADP-ribose residue, which is further involved in the synthesis of poly (ADP-ribose). Formation of this polymer is likely to be a signal of DNA damage, which is involved in the recruitment of repair proteins to the damage site [154]. It needs to be taken into account, that poly (ADP-ribose) is a potent glycation agent, which is able to form characteristic Amadori compounds in histones [158]. Taking into account the potential epigenetic role of this modification in humans [159], this aspect needs to be explored in plants, and specifically in seeds, as well.

The protection of mRNAs, which are synthesized in seeds at the late maturation stage and are translated when germination is initiated, plays a critical role in the success of germination [160]. Thereby, their length, secondary structure and specific motifs required for translation, need to be preserved [64]. In mature dry Arabidopsis seeds, about 10,000 individual mRNAs were annotated, whereas in rice this number reached 17,000. These RNA pools were mainly represented by the transcripts of the genes, responsible for primary metabolism and protein synthesis [64,161,162,163]. Early translation of mRNAs, stored during maturation, gives access to a rapid re-onset of seed metabolic activity during germination [163]. Among all proteins, stored in seeds during the desiccation period, those involved in translation can be expected to be more amenable to non-enzymatic modification during the period of dehydration, sub-sequent rehydration and germination [162]. In agreement with this, the proteins involved in translation were reported as particular targets of both protection and repair [162,164].

The loss of germination efficiency during natural or artificial ageing is accompanied by decreased contents and compromised integrity of mRNAs, which are highly amenable to oxidative damage by ROS [165]. The alterations in structures of mRNAs might result in translation blocks, whereas the loss of translational activity correlates with decreased seed germination rates [166]. The RNA repair system includes ATP-dependent RNA ligase, nucleotidyl transferases, and enzymes modifying the RNA ends for ligation (phosphatase and kinase) or protection (methylase) [167].

The protection of seed proteins from dehydration-related damage mostly relies on redox mechanisms. In this context, reversible formation of disulfide bonds in proteins plays the key role in protein protection during seed dehydration: disulfide bonding makes them more compact, allows attenuation of activity suppression, and protects against proteases [168]. Proteins are usually synthesized in a reduced sulfhydryl (SH) form and oxidized to yield more stable disulfide (S-S) form during seed maturation and dehydration [169]. After the completion of germination, the sulfhydryl form of seed proteins is restored by thioredoxins [170]. During seed formation and maturation, these proteins suppress the activities of the enzymes, catalyzing degradation of storage proteins and carbohydrates, and promote their re-activation afterwards—at the stage of germination [170]. 

Importantly, during storage, seed proteins accumulate spontaneous oxidative, glyco- and lipoxidative non-enzymatic covalent post-translational modifications [36,79,103,104,166,171,172]. Although these modifications are mostly irreversible, oxidation at some amino acid residues is often reversible [147,168]. This reversibility can be underlied by both enzymatic and enzyme-independent mechanisms [162]. For example, Châtelain et al. showed that methionine sulfoxide reductase (MSR) reduces methionine sulfoxide to methionine, contributing, thereby, to the maintenance of the dormant seed longevity in the model legume *Medicago truncatula* L. [168]. The loss of protein function can also result from the conversion of aspartate or asparagine residues into abnormal isoaspartic acid by spontaneous covalent modification. These abnormalities can be restored by *L*-isoaspartyl methyl transferase (PIMT) which repairs age-dependently damaged *L*-isoaspartyl and *D*-aspartyl residues in proteins via methylation by their side-chain carboxylic groups. *L*-succinimide, formed after a non-enzymatic rearrangement, can be hydrolyzed yielding *L*-amino acid residues. Overexpression of PIMT1 in Arabidopsis enhanced seed longevity, whereas reduced PIMT1 expression led to loss of seed vigor [67,147]. Long-term and inappropriate storage of seeds might result in enhancement of protein glycation, which is usually referred to as an array of non-enzymatic post-translational modifications formed by reducing sugars and carbonyl products of their degradation [36,79,127,173]. Conversion of carbonyl compounds in corresponding sugars and alcohols can be mediated by the activity of aldo-keto reductase family 1 (AKR1) enzymes [174]. Indeed, the over-expression of AKR1 was recently shown to improve seed longevity in tobacco and rice by detoxifying reactive carbonyl compounds generated during the storage-related aging. Small HSPs and LEA proteins, which ensure correct folding of synthesized proteins, protection from oxidative damage, and restoration of the original structure, can be also involved in repair of protein damages [19,31,162].

## 8. Loss of Desiccation Tolerance during Seed Germination

Based on the dynamics of water uptake and metabolic re-activation, seed germination can be divided into three phases [13,96,175]. Phase I (imbibition) is accompanied by rapid water uptake, hydration of macromolecules and repair of membrane, protein and DNA damage accumulated during the period of seed storage. This period is also characterized by activation of aerobic respiration in mitochondria. The phase II is characterized with a decrease of water uptake and enhanced mobilization of storage compounds. Seed metabolism is re-activated, that results in activation of protein biosynthesis. The end of the phase II is usually defined as the moment of radicle protrusion, which is caused by elongation and division of root cells. At this moment, the process of germination *sensu stricto* is finished and the post-germination phase III begins [13]. Interestingly, the orthodox seeds can preserve desiccation tolerance throughout the phases I and II, i.e., germination can be stopped during this period and imbibed seeds can be dried up to the initial dehydration level with a possibility of metabolism reactivation upon following rehydration [4,8,9,176]. This stage of germination, when reversible dehydration/rehydration cycles are still possible without loss of germination efficiency, is often referred to as the “window of desiccation tolerance” [176]. 

In this context, the transition from the phase II to the phase III (transition from seed to seedling) is the critical moment of the plant ontogenesis, which is accompanied with an irreversible loss of desiccation tolerance [9,149] (Figure 1C). Apparently, at this moment, the program controlled by the LAFL network is getting blocked [61,176]. The suppression of LAFL expression during seed germination relies on the homeodomain-containing proteins High-level expression of Sugar-Inducible gene 2 (HSI2) and HSI2-like1 (HSL1), which are also called VP1/ABI3-like1 (VAL1) and VP1/ABI3-like2 (VAL2) [61]. Modifications of chromatin, which involve the chromatin remodeling complexes—Polycomp Repressive Complexes 1 AND 2 (PRC1 and PRC2, respectively), as well as the PICKLE (PKL) and PICKLE-RELATED 2 (PKR2) proteins, represent the key mechanism behind the repression of the LAFL transcriptional network by the HSI2 and HSL1 proteins during seed germination. Recently, the chromatin remodeling factor PKL was also shown to block the expression of the *DOG1* gene directly [177].

## 9. Conclusions

The mature seed is a highly-tolerant organism, which is able to sustain extreme environmental conditions during prolonged storage. According to Chahtane et al., seeds transform plants into time and space travelers, which undoubtedly explains the success of angiosperms among terrestrial plants in colonizing numerous habitat [72]. Seed development can be divided in two main steps—embryo development and seed maturation. Early- and middle seed maturation stages are accompanied with accumulation of nutrients. At the late maturation stage, orthodox seeds develop desiccation tolerance, which allows maintaining seed viability after the loss of up to 95% of water and onset of dormancy. Desiccation tolerance allows long-term survival of dormant seeds under varying and often adverse environmental conditions. Thus, desiccation of seeds during maturation is a necessary event in the life cycle of the most of terrestrial plant species. Indeed, it underlies seed longevity, i.e., the ability of seeds to preserve their germination potential during prolonged times without detectable damage. The mechanisms behind the desiccation tolerance mostly rely on LEA proteins, small heat shock proteins, non-reducing oligosaccharides and antioxidants. Interestingly, orthodox seeds are tolerant to desiccation not only during the period of dormancy, but also in the germination phase, up to the moment of embryonic root initiation. Up to this time point, the seeds can be dried without loss of viability and the metabolic processes can be resumed upon sub-sequent re-hydration. The mechanisms behind this fascinating feature of germinating seeds are still not well understood. Therefore, it is necessary to identify genes, underlying the control of seed desiccation tolerance, and to understand the mechanisms behind their blockage during seed to seedling transition. It might provide a new insight in the problem of increasing drought tolerance in the plants developed from orthodox seeds.

## Abbreviations

ABA	Abscisic acid
ABI	ABA-INSENSITIVE
AGEs	Advanced glycoxidation end products
AGL	AGAMOUS-like
AHG	ABA Hypersensitive Germination
AKR	Aldo-ketoreductase
BER	Base excision repair
CMT	Chromomethylase
DME	Demeter
DOG	DELAY OF GERMINATION
DOGL	DELAY OF GERMINATION-like
DREB2D	Dehydration-responsive element-binding protein 2D
DT	Desiccation tolerance
DRM	Domains rearranged methyltransferase
FPG	Formamidopyrimidine-DNA glycosylase
FUS	FUSCA
GA	Gibberellic acid
GA3ox	Gibberellin 3-oxidase
GAAS	Germination Ability After STORAGE
HsfA	Heat shock transcription factor
HSI2	High-level expression of Sugar-Inducible gene
HSL1	HSI2-like1
HSP	Heat shock proteins
LAFL	Network of transcription factors (LEC1, ABI3, FUS3 and LEC2)
LEA	Late embryogenesis abundant
LEC	LEAFY COTYLEDONS
LPP	Lipid phosphate phosphatase
MET	Methyltransferase
MSR	Methionine sulfoxide reductase
NCED	9-cis-epoxycarotenoid dioxygenase
OGG 1	8-oxoguanine DNA glycosylase/lyase
PARP	Poly (ADP-ribose) polymerase
PIMT	*L*-isoaspartylmethyl transferase
PKL	PICKLE
PKR	PICKLE-RELATED
PLATZ	Plant AT-rich sequence- and Zinc-binding protein
PRC	Polycomb Repressive Complex
PYR/PYL/RCAR	Pyrabactin resistance/PYR-like/Regulatory component of abscisic acid receptor
RdDM	RNA directed DNA Methylation
RFO	Raffinose family oligosaccharides
ROS	Reactive oxygen species
ROS1	Repressor of silencing1
DML1	Demeter-like1
SMP	Seed maturation protein
SnRK	Sucrose-nonfermenting related kinase
TXN	Thioredoxin
VAL	VP1/ABI3-like
